# Understanding Rhinovirus Circulation and Impact on Illness

**DOI:** 10.3390/v14010141

**Published:** 2022-01-13

**Authors:** Camille Esneau, Alexandra Cate Duff, Nathan W. Bartlett

**Affiliations:** Hunter Medical Research Institute, College of Health Medicine and Wellbeing, University of Newcastle, New Lambton Heights, NSW 2305, Australia; camille.esneau@newcastle.edu.au (C.E.); alexandra.duff@uon.edu.au (A.C.D.)

**Keywords:** respiratory, rhinovirus, typing, circulation, serotyping, childhood, asthma, genotyping, virulence

## Abstract

Rhinoviruses (RVs) have been reported as one of the main viral causes for severe respiratory illnesses that may require hospitalization, competing with the burden of other respiratory viruses such as influenza and RSV in terms of severity, economic cost, and resource utilization. With three species and 169 subtypes, RV presents the greatest diversity within the Enterovirus genus, and despite the efforts of the research community to identify clinically relevant subtypes to target therapeutic strategies, the role of species and subtype in the clinical outcomes of RV infection remains unclear. This review aims to collect and organize data relevant to RV illness in order to find patterns and links with species and/or subtype, with a specific focus on species and subtype diversity in clinical studies typing of respiratory samples.

## 1. Introduction

One of the most common presentations of rhinovirus (RV) infections is described in lay terms as the ‘common cold’, with mild symptoms including a runny nose, congestion, sore throat, and cough. But this ‘common cold’ virus is not limited to the upper respiratory tract and, in the last two decades, RVs have been reported as one of the main viral causes for severe (lower respiratory tract) respiratory illnesses that may require hospitalization [1,2]. RVs are now established as a major cause of respiratory illnesses, and its most severe clinical presentations may not only be substantial, but also more costly than other common respiratory viral diseases including influenza or respiratory syncytial virus (RSV) [3,4,5]. Among more severe clinical presentations, RV is associated with exacerbations of chronic respiratory illnesses such as asthma and chronic obstructive pulmonary disease (COPD) exacerbations, chronic bronchiolitis [6] and community-acquired pneumonia (CAP) [7].

Clinical manifestations of RV infections are varied, and illness severity may be associated with a range of host and environmental factors. In addition, factors relating to the virus itself may play a role in tipping the balance between an asymptomatic infection and a severe illness. However, research on this topic is complicated by the fact that RV is the most diverse family of viruses within the enterovirus genera. The current RV classification includes three species (RV-A, RV-B, and RV-C) which are further divided into 169 subtypes, as listed on the Picornaviridae Study Group Subcommittee website [8,9]. Through November 2021, 80 RV-A, 32 RV-B, and 57 RV-C subtypes are described. Identifying the most clinically relevant subtypes among those may help develop targeted therapeutic approaches against this virus. Despite efforts, few links have been made between RV species/subtypes with symptoms, seasons, severity, and other viruses. More virulent RVs may be ‘hidden in plain sight’ due to the sheer number of subtypes in circulation in the population. This review aims to collate and update data describing RV transmission in relation to seasonality, at-risk populations, and other circulating viruses. The second part of this review will explore RV subtype diversity in published clinical studies to provide insight on RV circulation in relation to illness, revealing possible avenues for research to understand the virulence of specific RV species or subtypes.

## 2. Rhinovirus Epidemiological Factors

### 2.1. RV Transmission and Associated Illnesses

RV transmission is thought to occur through contact with fomites (virus-contaminated objects), or hand-to-hand contact followed by self-inoculation [10,11]. RV infectivity on surfaces rapidly declines, in a matter of hours [12]. Novel research, spurred by the COVID-19 pandemic, suggests that most respiratory viruses are also transmitted in an airborne manner (microdroplets referred to as aerosols) [13,14]. A third contamination route involves larger droplets and appears to account for 10% of contaminations following close contact with an infected individual (0.2 m or less) [15,16].

Following an incubation period of approximately two days, a symptomatic infection may occur [17,18]. The clinical presentation of RV infections is varied and ranges from mild upper respiratory tract illnesses (UTRI) to more severe lower respiratory tract illnesses (LRTI). In lay terms, URTI is generally referred to as ‘the common cold’, characterized by symptoms including nasal discharge, congestion, sore throat, and headache that does not usually interfere with breathing gas exchange/normal blood oxygenation [19]. Among the severe clinical cases, RV infection causes bronchiolitis [20,21] and CAP [22,23]. Both are defined by inflammation of the lung associated with severe cough, fever, and difficulties breathing/airway obstruction causing wheezing (a high-pitched whistling sound) that can lead to hypoxemia and respiratory failure. Frequent RV infections with wheezing during the first years of life can lead to the development of chronic respiratory illnesses such as asthma, defined by narrowing of the airways due to inflammation and constriction, resulting in shortness of breath, wheezing, and cough [24]. For individuals with chronic airway diseases, RV infections are the most common cause of exacerbations, characterized by a sudden worsening of symptoms often requiring treatment [25]. In addition to respiratory diseases, RV is associated with acute otitis media, an inflammation of the ear that is very common in children and a leading cause of hearing loss in developing countries [26,27].

### 2.2. Clinical Presentation and Symptom Severity

RV includes three species (RV-A, RV-B, and RV-C), and clinical studies of RV-positive subjects do not report a difference in terms of the clinical presentation associated with one or the other RV species [28,29,30,31]. Individual studies note differences in symptom presentation, but these are not sufficient to distinguish between RV-A, RV-B, or RV-C [32]. While symptoms are unable to discriminate between RV species, there is increasing evidence suggesting that RV-A and RV-C are not only more prevalent, but also more frequently associated with severe disease. In contrast RV-B is more frequently detected in asymptomatic subjects [31,33,34,35,36,37,38,39,40]. The capacity of a virus to replicate to a higher level is a potential determinant of pathogenicity. A higher viral load associated with more severe symptoms, including longer hospital stay, is frequently reported in clinical studies [36,41,42,43]. In hospitalized children, RV-C patients had a significantly lower PCR Ct value (which indicates higher level of viral RNA) in comparison to RV-A, suggesting a higher viral load for RV-C. This was associated with a significantly higher symptom score with RV-C species in comparison to RV-A [32]. Viral load alone may, however, not be sufficient to explain symptom severity as other studies do not report a higher viral load in association to RV illnesses requiring hospitalization [44]. Symptom presentation associated with RV infection is complex and may rely on a number of other factors such as co morbidities, co-infection, age, virus type and asthma phenotype [45]. Disease severity in relation to these factors will be discussed in the following sections of this review.

### 2.3. Rhinovirus Seasonality

RV infections occur year-round, however seasonality is characterized by a major peak of infection in the autumn/fall and a smaller one in the spring [46]. This seasonal pattern is supported by numerous studies investigating RV circulation in a clinical setting [47,48,49,50]. RV-A and RV-C are the predominant circulating species, swapping dominance throughout the year. RV-B is not as frequently detected but appears to alternate prevalence with other enteroviruses [31]. Peak seasonality of RV species may differ between RV-A and RV-C. Indeed, RV-C has been found to be predominant during the winter months while RV-A is the main species detected in the remainder of the year [51,52,53]. Studies assessing subtype diversity in Cambodia, in the Netherlands, and in Italy reported a predominance of RV-C in the winter months, while RV-A circulation in winter was minimal [49,54,55].

Viral illnesses depend on several factors that are external to the virus, notably seasonal variation (humidity, temperature, and climates). Seasonality may influence illness severity, as shown in a 2012 study reporting a predominance of RV species in autumn and spring, contrasting with an increase in RV illnesses during winter [33]. Ng et al. in 2018 studied RV circulation in Malaysia over three years and linked the data with local meteorological data. The peak of RV detection, around October and December each year, coincided with a high number of rainfalls and high humidity [36]. This agrees with epidemiological data observing that RV circulates more efficiently in the rainy season of tropical countries in Latin America [56]. Contradicting these findings, Lopes et al., observed an increased RV circulation in the dry season in Brazil, with infections peaking in November and December [57]. A study in Cambodia also showed that RV was predominant in the winter months which correspond to the dry season in this country [54].

RV is a globally circulating virus, with many RV sequences being found on several continents and communities at the same time [58,59]. As indicated by their seasonality and exchange in prevalence of RVA and C species, there is a rapid turnover in RV subtypes over a short period of time. These rapid changes are reported in clinical studies using typing and emphasize the circulation of RVs over large geographical areas. For example, a Kenyan study reported the detection of the same RV subtype in communities 30 km apart, indicating a rapid RV spread in the population [50]. Similarly, RVs are prevalent even in communities that are geographically isolated, as illustrated by a multicentre study in Colombia which examined RV diversity in increasingly isolated villages [60]. The COVID-19 pandemic once again showed omnipresence of RV subtypes and their ability to circulate in conditions that are not favourable to other respiratory viruses. The non-pharmaceutical measures used to curb SARS-CoV-2 transmission (mask wearing, social distancing, lockdowns, etc.) resulted in a drastic reduction in incidence of respiratory viruses including influenza, adenovirus, and RSV. On the contrary, RV stood out and appeared to cocirculate with SARS-CoV-2 at a similar rate to its habitual seasonal patterns, despite the reduction in contacts due to the public health measures [61,62]. A higher infectivity and prevalence in children, who are less subject to social distancing measures and mask wearing, may explain the lesser impact of public health measures on reducing RV transmissions in comparison to other respiratory viruses [63]. RV infections in the context of co-infections with other respiratory pathogens will be discussed later in this review.

### 2.4. RV Treatment Strategies

There are no approved preventative therapies or vaccines for RV, and current treatments focus on reducing host response-mediated symptoms rather than targeting the virus to limit replication and duration of infection. Early vaccination trials were encouraging and showed that an immune response against RV could be protective against reinfection [64,65,66]. Unfortunately, the lack of cross reactivity between RV subtypes hindered progress and underlined RV antigenic diversity as the major challenge for vaccine development [67,68,69]. In 2016, Lee et al. demonstrated that a cross-reactive RV vaccine could be possible by showing neutralization of 50 RV subtypes with a single polyvalent vaccine in rhesus macaques [70]. As targeting a third of all RVs subtypes can be achieved, identifying clinically relevant subtypes, notably through increased surveillance of circulating RVs, would be the next step required to direct vaccine development [71].

Development of antivirals against RV infections has been the aim of numerous studies (summarized in multiple recent reviews [72,73,74,75,76,77]). Amongst early antiviral strategies, virus-targeting capsid-binding drugs (Pleconaril, Pirodavir, Vapendavir) showed efficacy on preventing virus entry [78,79], but posed issues of rapidly emerging drug resistance [80,81,82]. Other viral targets include conserved regions in the viral RNA-dependent RNA polymerase and viral proteases (rupintrivir), but no drug has achieved approval for clinical use [83,84].

The emergence of novel respiratory viruses such as SARS-CoV-2 has further highlighted the importance of identifying broad spectrum antivirals. Host innate-immune directed antiviral strategies offer the potential to work against multiple respiratory pathogens. Such therapeutics include antimicrobial molecules which can directly act as effectors against the virus, such as cathelicidins, lactoferrin, or vitamin D [85,86,87,88]. Recently, a defensin-like peptide (P9R) was reported to be effective against many pH-dependent viruses including RV, coronavirus (SARS-CoV, SARS-CoV-2, and MERS-CoV), and influenza viruses (H1N1, H7N9) [89].

Interferons (IFN) are another class of antiviral innate immune-stimulating molecules that have been studied extensively. Early research tested the prevention of severe RV symptoms by prophylactic intranasal alpha 2 IFN treatment. Treatment did reduce virus-induced symptoms, however this was offset by IFN-induced nasal inflammation [90]. Inhaled nebulized IFN-β was tested in an asthmatic cohort to determine if it can prevent viral exacerbations. Despite not reaching its primary endpoint, this research showed the potential of modulating innate immune responses in susceptible populations [91]. Recently, use of nebulized IFN-β for the treatment of hospitalized COVID-19 patients showed clinical benefit by reducing the risk of progressing to severe disease [92]. Rather than using individual innate antiviral effector molecules, an alternative approach is the activation of innate immunity via stimulation of pattern recognition receptors such as Toll-like receptors (TLRs). Cell surface TLRs such as TLR2 are key regulators of airway mucosal innate immunity and RV [93], and SARS-CoV-2 [94] structural proteins activate TLR2. Innate immune priming with a synthetic TLR2/6 agonist has been shown to protect against RV [66], influenza [95], and SARS-CoV-2 [96].

## 3. RV Infections in At-Risk Populations

### 3.1. Children

Children are the main reservoir for RVs and support the transmission of RVs in the community, having up to four times more infections a year than adults [97]. Contact with other children and adults may be the most important source for circulation of RV subtypes in the community. Illustrating this, the return from holidays/back-to-school season in the Northern hemisphere correlates with peak RV seasonality, an increase in emergency department admission, and asthma exacerbations [98,99,100]. RV-positive cases decrease with age [42,50]. Beyond mere frequency of infection, there is an increased risk of developing a symptomatic infection at a young age. This was exemplified in an African case-control study, where RV infection was more often associated with pneumonia for children between 13 and 59 months old [34]. Another study focusing on hospitalized children reported that 71.4% of all RV severe acute respiratory tract infections were found in children less than three years old [32]. Finally, most RV-positive cases in Tunisian children hospitalized with severe acute respiratory infections (SARI) were detected in those less than six months old [101].

RV-C species seem to have a particular association with illness detected at a young age and more frequently isolated in children under two years [29]. On the other hand, RV-A is more frequently observed in adult populations [102]. This increased susceptibility to RV-C could be due to an immaturity of the immune system of young children, with RV-A becoming more common with age due to its overall higher prevalence. A recent pooled study using 17,664 samples from 14 cohorts from the United States, Finland, and Australia reasserts the specific association of RV-C infection with children under five years old. Using samples from the COAST (Childhood Origins of Asthma), Choi et al. also reported significantly more neutralizing antibodies responses against RV subtypes belonging to RV-C from two years of age and increasing with age. This indicates that RV-C is more immunogenic than RV-A, supporting the hypothesis that increasing protective immunity to this species with age allows RV-A infections to become increasingly prevalent [38].

### 3.2. Chronic Airway Diseases: Asthma

Relationships between RV and asthma have been discussed in numerous reviews [103,104,105]. RV infection is associated with hospitalization in the first year of age and is a predictor for recurrent wheezing, which is a symptom of a narrowed airway or inflammation [106]. RV-induced wheezing is very prevalent among children under five and is associated with asthma diagnosis at a later age [24,107]. Association of RV infections with asthma onset has been found to be true even in cohorts looking at children with low risk of developing asthma [108]. Not only relevant for asthma onset, RV has been found to be a trigger for exacerbation in children and adults after the establishment of the asthma diagnosis [25], with RV infections preceding as much as 50% of asthma exacerbations [109].

When RV-C species was discovered in 2006 [52,110], several studies aimed to determine whether RV-C species was more associated with asthma onset or exacerbations in comparison to RV-A. RV-C species have been associated with a higher number of asthma-detections and possibly increased asthma severity, notably in children under five years of age [45]. Other studies did not identify an association of RV species with chronic respiratory illness status or with clinical outcomes, admission to intensive care unit (ICU), or length of hospital stay [30,102]. RV-A may even be more clinically significant in asthmatic patients that do not require hospitalization, as it was associated with worse asthma symptoms and longer cough [111]. Therefore, the association of RV-C with asthma seen in early studies may be more associated with the age or increased susceptibility of the host to RV infection, such as CDHR3 polymorphism, rather than to an increased virulence of this species [38,112,113].

### 3.3. Elderly and Immunocompromised

The elderly is another group where RV infections are more common. RV is frequently detected in older patients that are arriving at the emergency department [114]. In addition to being frequently detected, older adults may be at a higher risk of severe complications. RV-associated pneumonia has been shown to have an increased morbidity and mortality rate in elderly patients when compared to pneumonia caused by influenza. Elderly patients with RV-associated pneumonia were also more likely to have a chronic respiratory illness, highlighting RV as a virus particularly associated with vulnerable populations [115]. Immunocompromised subjects may be particularly at risk of illness linked to RV infection [116]. A lack of immunity can lead to the development of chronic viral infection where rhinovirus cannot be cleared, leading to prolonged virus shedding and re-infection, possibly associated with the emergence of new RV subtypes [28,117].

## 4. Rhinovirus and Co-Infections

Another difficulty when trying to understand the RV relationship with disease is co-infection with common viral and bacterial respiratory pathogens. Clinical studies typing RV in respiratory samples report high rates of viral, fungal, or bacterial co-infection, as high as 78.9% [118,119,120]. Dual infections may be linked to more severe illnesses, particularly in immunocompromised populations [32]. Looking at RV circulation alone may not always inform on illness severity, and RV infections need to be considered in relation to other pathogens.

### 4.1. Bacterial Co-Infections

Bacterial co-infections could be a major factor associated with the severity of RV symptoms and hospitalization. RV infection and the host immune response to infection may promote bacterial proliferation resulting in a more severe illness. In recent clinical studies, co-infection was associated with a longer hospital stay in comparison to RV alone. Similarly, bacterial co-infection was associated with more severe illness in patients infected with either RV-C and RV-A species [30]. RV-A-positive hospitalized children could be more likely to develop a complicated infection when co-infected with bacteria [101]. Moreover RV/*S. pneumoniae* is the main viral-bacterial association reported in children hospitalized with CAP [121]. Similarly, Linder et al. found that bacterial co-infections caused increased lower respiratory compromise among RV-C cases [122]. During experimental RV-A16 infection, bacterial co-infection was associated with prolonged lower respiratory symptoms and exacerbation in COPD patients. Degradation of antimicrobial peptides by RV may favor secondary infection by bacteria [123]. Bacterial co-infection may finally play a specific role in the development of illnesses requiring hospitalization in adults. In their study focusing on hospitalized patients with severe acute respiratory symptoms, Hung et al. reported that adults had more frequent dual infections with bacteria, notably *Pneumocystis jirovecii*. The same study reported a higher rate of viral co-infection in RV-positive children [124].

### 4.2. Viral Co-Infections

RV co-infection with other respiratory viruses such as RSV, adenovirus, coronavirus, and parainfluenza viruses is frequent [125]. Like bacterial co-infection, infection with two or more respiratory viruses may increase illness severity. Indeed, they have been associated with an increased risk of ICU admission in comparison to single infections [126]. Viral co-infection may be particularly associated with a young age, as illustrated by an Australian study following children and adults with acute respiratory illnesses over a one-year period. The majority of viral co-detection occurred in children under two years of age, while no co-infections were found in RV-positive samples from patients over 14 years of age [31]. RV and RSV are both important contributors to childhood respiratory illnesses, and therefore dual infection with these pathogens has been studied. RV is one of the most common viruses detected in RSV-positive samples [126,127]. RV association with allergic sensitization might be enhanced in dual infections with RSV, and RV/RSV dual infections have been associated with recurrent bronchial obstruction and wheezing, leading to allergic sensitization [128,129,130].

While multiple viruses are often detected in respiratory infections, the association between dual infection and severity of illness may not be as straightforward as for bacterial co-infections. This can be exemplified with influenza: Even though mathematical models indicate that influenza/RV could be associated with more severe illnesses [131], some studies have reported that RV/influenza co-infections are occurring less often than expected [31]. Finally, there is evidence that RV peak seasonality in autumn resulted in a delayed 2009 H1N1 epidemic in Europe [132,133]. It has been proposed that infection by RVs may interfere with other respiratory viruses and promote viral clearance. This concept of viral interference will be discussed in the following section.

### 4.3. Viral Interference—RV Protection from Viral Illnesses Caused by Other Respiratory Viruses

While there are multiple clinical scenarios in which RV can cause severe disease, there is also evidence that a primary RV infection can protect from subsequent infection and disease caused by other respiratory viruses.

RV is one of the main viral pathogens detected in association with SARS-CoV-2 cases and mathematical models suggest a protective role of RV circulation against more severe SARS-CoV-2 infection outcomes [134]. This is supported by experimental data, in which staggered infection of RV-A16 and SARS-CoV-2, regardless of order, showed a reduction in infectious titres in human bronchial epithelial cells differentiated at the air–liquid interface (ALI pBECs) [135,136,137]. RV infection, by stimulating innate immunity in the upper respiratory tract, may provide a temporary resistance to infection with SARS-CoV-2, as early innate immune response may be critical in limiting infection [138]. This was explored in a study using experimental staggered infection of RV/SARS-CoV-2 in an airway epithelial cell organoid model. Infection with RV-A1 three days before infection with SARS-CoV-2 resulted in elevated interferon stimulated gene (ISG) expression, and significantly reduced SARS-CoV-2 viral load after 72 h. This reduction in SARS-CoV-2 viral load was not observed when blocking ISG pathways [139]. RV infection increased expression of dACE2, an isoform of ACE2 (SARS-CoV-2 receptor for entry [140]). Previous airway epithelial cell expression studies showed that RV-A16 increased the expression of molecules (ACE-2 and TRMPRSS2) required for SARS-CoV-2 cell binding and entry, raising concerns that RV infections could promote SARS-CoV-2 infection [132,141]. However, this truncated isoform of ACE2 lacks the SARS-CoV-2 binding sites of the full-length isoform, and therefore is not expected to enhance infection [142,143]. Evidence of RV interference with other viruses has been demonstrated in two separate mouse studies. Mice were challenged first with RV, followed two days later by a lethal infection with influenza A virus (PR8) or pneumonia virus of mice (PVM). Exposure to RV-A1 prior to secondary viral infection was protective. The mechanism behind this phenomenon involved IFN-mediated early suppression of viral replication as well as downstream modulation of immune responses [144,145]. These results are further supported by an experiment in differentiated primary airway epithelial cells, where RV-A1 infection followed by influenza resulted in a 50,000-fold decrease in viral load when compared to virus alone [132]. In contrast, Essaidi-Laziosi et al. found that a clinical strain of RV-A16 was not able to interfere with infection by influenza virus or RSV in differentiated primary airway epithelial cell cultures but confirmed the importance of innate antiviral pathways (IFN) in viral interference [146]. A possible explanation for this discrepancy could be the virus used in each of these studies. Essaidi-Lazziosi et al. utilized the major group virus RV-A16, while previous studies used the minor group virus RV-A1. Minor and major group RVs have important differences, notably utilization of different receptors for entry [135,136,137]. Therefore, the mechanisms behind viral interference could be different between RV subtypes. Inter-subtype differences remain relatively unexplored and might influence conclusions relative to RV virulence. The next section of this review will explore subtype diversity in the literature, with a special focus on subtype detection in studies.

## 5. Beyond RV Species: A Focus on RV Subtype Circulation

Determinants of RV illness include a complex combination of host and environmental factors. These factors have long been a major focus to understand illness linked to RV infections, often overlooking the diversity within RVs. RV-A and RV-C species dominate over RV-B, both in general prevalence and in disease, but these two species alone encompass 137 of the 169 RV subtypes currently recognized. As more researchers call for an increased surveillance of non-influenza respiratory viruses, a major question remains regarding how RV diversity impacts clinical presentations. Identifying more prevalent subtypes, or subtypes associated with an increased risk of severe illness, may be important to develop preventative treatment approaches, including vaccine development [29,69,71].

### 5.1. Rhinovirus Classification System and Surveillance

PCR assays to detect RV have been used since the 1990s [147,148] but have seen considerable improvement over the years [149,150]. The increased accessibility of sequencing and typing has facilitated RV detection. This led to the discovery of RV-C, and eventually to a 2010 proposal to classify RVs based on their genetic homology (subtypes) rather than by their serum reactivity (serotypes) [52,58,110,151]. Using sequence identity thresholds for type assignment rather than physical and chemical properties of the virus has contributed to the facilitation of studies aiming to understand RV diversity in clinical respiratory samples. Nasopharyngeal aspirates or swab samples are used in diagnostic tests to detect the presence of an array of viruses [152]. RV species and subtype typing can be performed using PCR assays targeting mostly VP1 and/or VP2/VP4 genomic regions. These regions are very variable between subtypes, which makes them predictive of type. Under the current classification, a RV sequence belongs to A, B, or C species if it has at least 70% similarity with other subtypes within this species. Two RV sequences with >90 nucleotide identity on VP2/VP4 and >87% on VP1 regions (respectively) belong to the same subtype. A clinical RV isolate presenting with a lower sequence identity may indicate a new subtype, which can later be added to the virus classification by ICTV subcommittee after full sequencing of the VP1 region [153,154].

### 5.2. Understanding RV Subtype Diversity Using a Meta-Analysis Approach

In 2019, we published a review where we identified 23 clinical studies that reported RV subtypes, following their isolation in respiratory samples. Using this panel of published studies, we aimed to assess RV diversity in clinical samples. For each RV subtype, we counted the number of studies in which it was detected. This approach revealed that only a handful of RV subtypes were detected across half of the 23 clinical studies selected. RV-A12 and -A78, -B6 and -B27, -C15 and -C2 were reported in most studies for A, B, and C species, respectively. This suggested a persistence of these subtypes in the population, which could represent more clinically relevant subtypes. However, this initial survey of RV diversity in clinical studies presented limitations, notably because it did not consider the size of each study and did not compare A, B, and C species simultaneously [155].

In the context of this review, we sought to address some of these limitations, with the objective to highlight the main trends in RV diversity, circulation, and its association with illness through a ‘meta-analysis’ approach. We used a constructed PubMed search to identify in a systematic manner the studies performing RV typing on respiratory samples. This search included several keywords as follows: ‘(((((((Rhinovirus diversity) OR Rhinovirus phylogeny) OR Rhinovirus genetic diversity) OR Rhinovirus typing) OR Rhinovirus subtype) OR Rhinovirus molecular typing) OR Rhinovirus genetic diversity)’. The keyword ‘rhinovirus genotyping’ was subsequently added to this search. Search results were first screened based on title, and then individually reviewed for eligibility. Studies were selected if they were published after 2013 and when they performed typing on VP2/VP4 and/or VP1 on clinical respiratory samples, listing RV subtypes from all three RV species. In each individual study, we reported the number of times each RV subtype was detected. To account for the different sample size in each study, the number of detections was then divided by the total number of sequences typed in each study. Finally, subtype detection was expressed as a percentage over the total number of detections for all studies.

Our initial PubMed search returned 729 results as of November 2021, and 127 studies were selected for further review based on title. After review, 96 studies were excluded from the meta-analysis. Reasons for exclusion were as follows: not all RV species were typed, subtypes were not disclosed (37 studies), studies were published before 2013 (34 studies), studies were not clinical studies (20 studies), not published in English (5 studies) or the dataset was redundant with another clinical study included in our meta-analysis (3 studies). As a result, 31 studies typing RVs from all three species in clinical samples were included in the final analysis (Table A1, Appendix A).

### 5.3. Rhinovirus Species and Subtypes in a Panel of 31 Studies

The details of the 31 studies included in this analysis are presented in Table A1. Studies were published between 2013 and 2021, and covered samples from 26 locations which were collected between 2001 and 2006. Overall, the RV species distribution was fairly conserved between studies, independent of geographical regions and selection criteria. RV-A species was the most detected overall (56% on average—minimum of 44% of detection and a maximum of 75.9% of detection). The next most detected species was RV-C, with a minimum of 20% of subtypes belonging to this species and a maximum of 55% (the average was 34.5%). Although 19% of RV subtypes belong to RV-B species, the maximum of detection was 18.2% in one study and 8.5% on average for this species, consistent with the report of RV-B underrepresentation.

**Figure 1:**
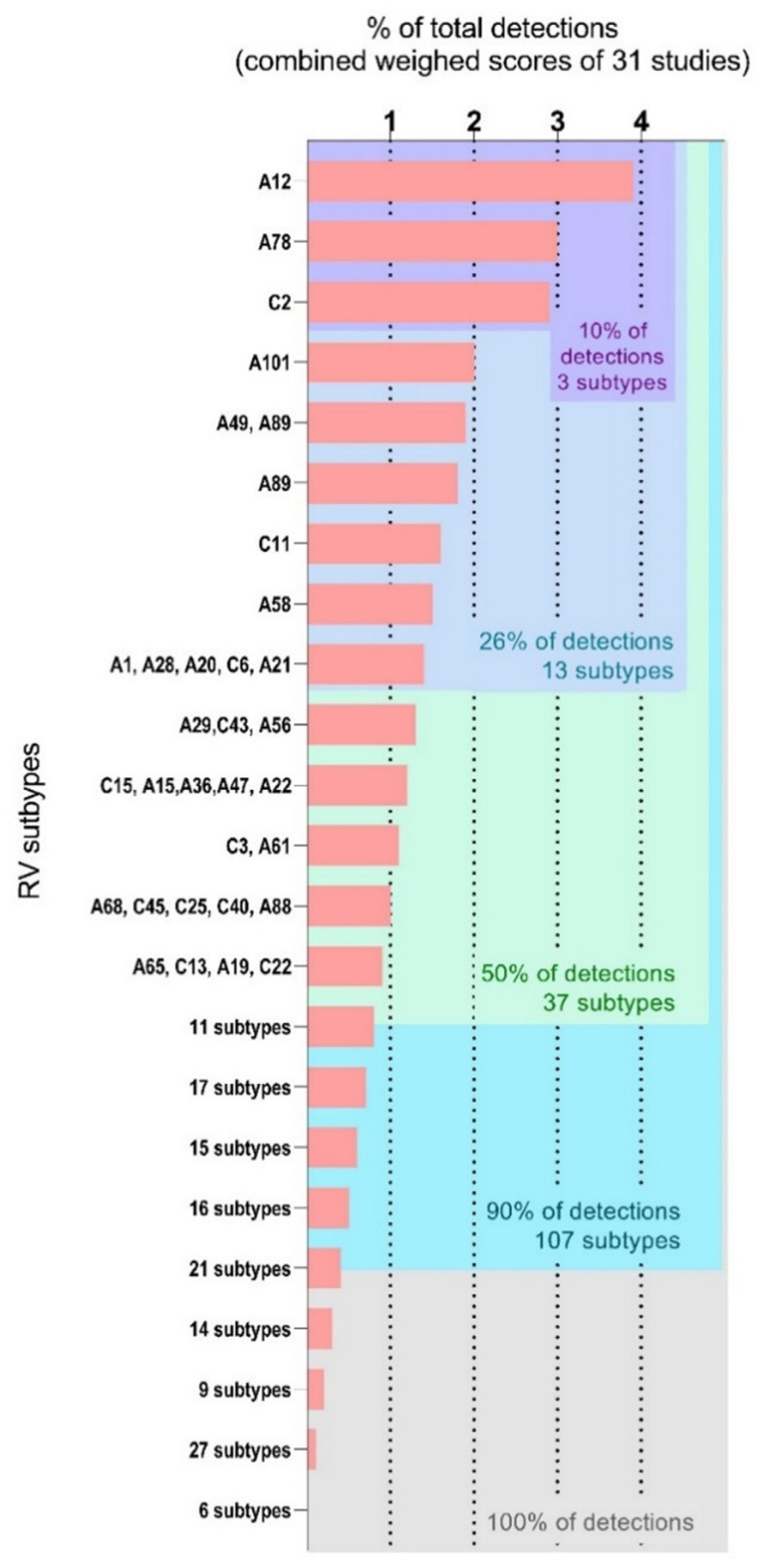
RV subtype contribution to total detections in a panel of 31 clinical studies typing RVs. RV subtypes are shown ranked highest to lowest for their contribution of total RV detections in the panel of 20 studies. When several subtypes are listed together, they each contribute to the same percentage of total detections.

Figure 1 shows RV subtype distribution in the panel of 31 studies, expressed in percentages of total detections. Three subtypes (less than 2% of all RVs), RV-A12, A78, and -C2, represented 10% of total detections. In comparison, the 56 lowest ranked subtypes (33% of all RVs) also amounted to 10% of all RV detections in this panel of studies. Within the 50% most represented RVs, 24 subtypes belonged to RV-A species and 13 to RV-C species. There was no RV-B present among the subtypes most often detected in clinical studies. Subtypes underlined in our meta-analysis approach were also described specifically in individual studies included in our panel. In 2013, Sansone et al. reported a predominance of RV-A78, -A49, -C12 in RV-positive samples from hospitalized children under five years in Sweden [156]. In their 2016 study typing 745 samples sent to diagnostic between 2007 and 2012 in the Netherlands, Van der Linden et al. identified RV-A12, -A78, and -C2 as the predominant subtypes. RV-A12 was noted to occur in clusters while -C2 and -A78 were detected over multiple years [49]. Similarly, Zhao et al. reported RV-A12, -A78, -A89, -C2, and -C6 among the predominant subtypes detected among 280 respiratory samples obtained from children with a respiratory illness between 2013 and 2015 [53]. At the same period (2014–2017), Andres et al. typed 1771 RV-positive samples obtained from patients with a respiratory tract infection suspicion. Once again, RV-A78, -A22, and -A49 and RV-C2, -C15, and -C12 were the most prevalent RVs over this period [157]. Clinical studies focusing on a smaller number of samples also reported RV-A12 as very prevalent over multiple seasons [158]. RV-A101 ranked fourth in our meta-analysis and was also mentioned as one commonly recurring subtypes [101,159].

These observations are in agreement with additional studies describing high occurrences of genotypes RV-A78, -A12 and -C2, once again suggesting an increased circulation of these subtypes. These studies were not included in our meta-analysis panel because they did not fit our selection criteria [160,161,162]. A higher prevalence does not necessarily mean a greater link with illness, but this relationship was investigated in the recent publication from Choi et al. who compared RV sequences obtained from multiple respiratory cohorts in the US. Their methodology allowed them to compare subtype occurrence in relation to illness status (well versus sick) and this revealed that RV-A12, -A78, and -C2 were not only more predominant, but were also associated with a higher proportion of illness, hinting at greater virulence [38].

### 5.4. Insight of Study Design on Questions Related to RV Subtype Circulation

Our meta-analysis illustrated RV broad circulation and sheer diversity. All but six subtypes (RV-A50, -A74, -C52, -B5, -B100, -B106) were detected in the panel of 31 studies, even though these studies are varied in terms of study design, location, and symptoms associated with the respiratory sample studied. The studies included in this analysis highlight the epidemiological factors that are at the centre of focus when aiming to comprehend RV circulation and virulence. Most studies came from countries in Asia (11 studies), eight looked at respiratory samples from European countries, and seven studies were located in African countries. This demonstrates the global presence of RVs and emphasizes the efforts of the research community in understanding RV subtype circulation all over the world. Fifteen out of the 31 studies included in our panel were conducted in samples from children exclusively. Furthermore, 10 out of 11 studies that used respiratory samples from both children and adults had low median age (ranging from 1.5 years to less than 10 years old), and only one study observed a mix of children and adults with a high median age (38 years). Only five studies were conducted in adults exclusively (median age ranging from 31 years to 77.5 years). This dominance of studies using samples from young populations illustrates the fact that RV is mainly associated with illnesses in childhood. Finally, studies selected in our dataset tended to look at samples linked to symptomatic infections, with the main selection criteria being acute respiratory illness (ARI), severe acute respiratory illness (SARI), or influenza-like illness (ILI). Only two studies were focused on asthma and COPD exacerbations, despite the frequency of association of RV with these complications. Looking at hospitalization status, 12 studies used hospitalized patients’ samples and seven studies focused on outpatients. Seven studies were a mix of hospitalized patients and non-hospitalized patients, with only two of them providing a breakdown of RV types according to the hospitalization status [34,49]. Hospitalization status in relation to RV subtype was not clearly stated in five studies. This indicates that RV contribution to total detections illustrated by Figure 1 reflects the representation of subtypes in illnesses, but also illustrates the lack of information available on subtype diversity in asymptomatic infection or in the context of chronic illnesses.

To try and understand the influence of geographical regions on RV subtype circulation, we compared the rankings of subtypes in the subsets of studies from Asia, Europe, and Africa to the overall ranking. This comparison is shown in Table 1 and revealed potential subtype differences related to geographic distribution. While RV-A12, -A78, and -C2 remained the highest contributors to total detections all three those subsets, some RVs rankings was influenced by the geographical region. For example, RV-A43 appeared to be underrepresented in clinical studies typing samples from Asia, as it did not appear in the 25 highest ranked subtypes for this subset but was highly ranked in European and African studies subsets. Interestingly, while no RV-B subtypes were found in the top contributors to detections in the general ranking, RV-B69, -B70, -B48, and -B84 appeared in the top 25 subtypes in the subset of studies from Africa. This may indicate a higher prevalence of these subtypes in Africa, or highlight an increased vulnerability to RV-B species, generally regarded as less pathogenic. We finally compared RV rankings between studies using samples from hospitalized patients to the general subtype ranking, and to the subset of studies using outpatients only (Table 2). RV-C9 gained 85 places in the hospitalized subset ranking when compared to general, possibly suggesting a particular association with illness requiring hospitalization. Differences in ranking between the hospitalized and outpatient study subset revealed even more RV types that were ranked higher in the hospitalization subset. Therefore, these subtypes should be further investigated in their association to illness. While study breakdowns could point towards a differential RV prevalence in geographic regions or in association to severe illness, these observations need to be considered with caution, particularly when comparing rankings in smaller subsets of studies as this could introduce significant bias.

### 5.5. Challenges in Understanding RV Subtype Circulation and Link to Illness

As illustrated in this review, the clinical outcomes resulting from RV infections are likely to be associated with factors external to the virus, such as age, seasonality, meteorological factors, and co-infections. The diversity of rhinovirus subtypes, their very high prevalence, and the range of symptoms they can create makes identifying virus-specific links to illness an arduous task, more so than for other respiratory pathogens (influenza, coronaviruses).

Typing of clinical isolates in studies is usually based on sequencing of one region of the RV genome (e.g., 5′UTR, VP2/VP4, or VP1). While these regions are predictive of type, they may occasionally result in contradicting subtype attributions [54,163]. Although molecular assays are constantly improving, some RV subtypes with significant divergences within these genomic regions may remain undetected by those assays. Finally, the occurrence of recombination or point-mutations may result in antigenic divergences on the protein level, as reported by studies comparing genotyping with serotyping methods [119,164,165,166]. These differences could play a role in virulence which might not be apparent when typing RVs. Therefore, typing methods might not paint a complete picture of the properties of clinical isolates and of their association with illnesses.

By reviewing the subtypes in published clinical studies, we found only a handful of RV subtypes were associated with the majority of detections reported in the past two decades, despite the considerable variations in geographical regions, sample selection criteria, and year of sampling within our selection of studies. Comparison of studies subsets revealed a differential geographic distribution and association with hospitalizations. Further investigation is required to confirm the clinical relevance of these RVs. Understanding clinical relevance may be crucial, particularly to inform laboratory research, as the body knowledge on RV pathogenesis is concentrated on models (such as RV-A1, RV-A16 and RV-B14) that likely differ from subtypes relevant in illnesses. Finally, it is important to note that our ‘meta-analysis’ approach may not be the best to investigate subtypes diversity, as we centred it solely on published studies reporting typing. Although a systematic approach was used to find those studies, some could have been accidentally omitted. Analysis of pooled clinical datasets with specific association with illness presentation, such as reported by Choi et al., may be more informative and reliable to draw conclusions [38]. 

## 6. Conclusions

This review has explored the complex relationship between RV and disease. Despite this complexity, some clear signals have emerged indicating that different RV species do play a role in specific disease scenarios. Certainly, RV-A and RV-C are more prevalent and associated with more severe illness outcomes. These two species could exhibit differences in terms of seasonality, as they have been reported to alternate in dominance over months. The prevalence of RV species might be predominantly influenced by age, with data suggesting that RV-C may be particularly relevant in early childhood. Specific association of RV species or subtypes with illness severity remains unclear, as illustrated in the case of asthma, the elderly, and immunocompromised individuals. Finally, RVs co-exist with many other respiratory pathogens and are frequently associated with multiple infections of bacteria or viruses. If bacterial/RV co-infections appear to be linked to more severe clinical presentations, this might not always be the case when looking at viral/RV co-infections. Indeed, milder RV infection may have a protective effect against other respiratory viruses, including influenza and SARS-CoV-2 via stimulation of innate anti-viral immunity in the upper respiratory tract. Organizing a better surveillance of RV species and subtypes is crucial if we want to understand the intricate variables that determine disease outcomes following RV infection. With studies typing RVs in a large number of respiratory samples, clinically relevant patterns connected to specific RV subtypes may emerge, as hinted here by our meta-analysis on 31 clinical studies. A handful of RV subtypes (including RV-A12, -A78, and -C2) appear to be more prevalent, and further research is required to confirm this and understand why. Identifying the most clinically relevant RVs (across life stages and in different clinical scenario) will provide a better understanding of RV circulation and disease, benefit basic translational research to develop clinically relevant models of infection, and later allow the development of targeted treatments, particularly for populations most vulnerable to RV infection-induced diseases.

## Figures and Tables

**Table 1:** Influence of geographical region on subtype rankings. Ranking of subtypes in the subset of studies was compared to the general ranking (31 studies). A positive number indicates that the subtype is ranked higher in the analysis subset than in the general ranking. Subtypes gaining more than 40 ranks are highlighted in green. Number of studies in each subset is indicated between brackets.

Studies from Asia (11)			Studies from Europe (8)			Studies from Africa (7)		
Top 25 Subtypes	% Detections	Rank Compared to General	Top 25 Subtypes	% Detections	Rank Compared to General	Top 25 Subtypes	% Detections	Rank Compared to General
A12	4.1	0	A78	4.8	0	A12	4.7	0
C2	3.0	1	A12	4.3	1	A101	3.6	2
A78	2.5	−1	C43	2.8	12	C2	2.9	0
A49	2.4	1	C2	2.8	1	A78	2.8	−2
A89	2.2	1	A49	2.2	1	C13	2.4	25
A36	1.9	13	C25	2.1	13	A65	2.2	23
C6	1.9	5	A22	1.9	5	A20	2.1	4
A56	1.9	8	A28	1.8	8	A15	2.1	10
A21	1.9	4	C5	1.8	4	C45	2.1	16
A47	1.7	10	A59	1.7	10	A28	1.9	0
A29	1.6	3	A58	1.7	3	A58	1.9	−3
A61	1.6	11	A89	1.7	11	C11	1.8	−5
A68	1.5	11	A101	1.7	11	A47	1.7	7
C3	1.4	8	A56	1.7	8	A1	1.7	−5
A38	1.4	47	C6	1.6	47	A2	1.7	32
C1	1.3	21	C15	1.6	21	C43	1.6	−1
A20	1.3	−6	C22	1.4	−6	C22	1.6	15
C11	1.3	−11	A21	1.2	−11	B69	1.5	62
A34	1.3	20	A53	1.2	20	B70	1.5	52
A1	1.3	−11	C12	1.1	−11	A81	1.5	37
C15	1.3	−4	C3	1.1	−4	B48	1.4	39
A7	1.3	27	A10	1.0	27	C36	1.3	54
A33	1.2	18	C7	1.0	18	C3	1.2	−1
C35	1.2	34	C23	1.0	34	B84	1.1	59
A101	1.2	−21	C27	1.0	−21	A29	1.1	−11

**Table 2:** Influence of hospitalization status on subtype rankings. Ranking of subtypes in the hospitalized subset was compared to the general ranking (31 studies) and to the subset of studies focusing on outpatient. A positive number indicates that the subtype is ranked higher in the hospitalized subset than in the general ranking. Subtypes gaining more than 40 ranks are highlighted in green. Number of studies in each subset is indicated between brackets.

Hospitalized (12)			Hospitalized vs. Outpatients		
Top 25 Subtypes	% Detections	Rank Compared to General (31)	Top 25 Subtypes	% Detections	Rank Compared to Outpatients (9)
A12	6.0	0	A12	5.4	0
A78	5.0	0	A78	4.8	1
C2	3.1	0	C2	4.0	−1
A101	2.8	0	A101	2.9	12
A89	2.2	1	A89	2.6	87
C6	2.1	6	C6	2.1	13
A61	2.1	16	A61	2.1	92
A22	2.0	13	A22	1.8	118
C43	2.0	6	C43	1.8	71
A49	1.9	−5	A49	1.7	17
A56	1.7	5	A56	1.7	21
A88	1.7	16	A88	1.5	51
A68	1.6	11	A68	1.5	67
A36	1.6	5	A36	1.4	15
C25	1.5	11	C25	1.4	21
A20	1.5	−5	A20	1.4	−9
A15	1.5	1	A15	1.3	−2
C45	1.4	7	C45	1.3	95
A59	1.4	16	A59	1.3	50
C9	1.3	85	C9	1.3	28
A28	1.3	−11	A28	1.3	−15
A80	1.3	32	A80	1.2	42
A16	1.2	30	A16	1.2	38
A58	1.2	−16	A58	1.2	−19
C22	1.2	13	C22	1.2	−14

## Appendix A

**Table A1:** Details of clinical studies used in the final meta-analysis. Abbreviations: ILI: influenza-like illness, ARI: acute respiratory illness, SARI: severe acute respiratory illness, RTI: respiratory tract infection, LRTI: lower respiratory tract illness, URTI: upper respiratory tract illness; EU: European Union, CAP: community-acquired pneumonia, ICU: intensive care unit. * Total number of samples not specified ** The number of occurrences for each subtype was not disclosed.

Study Reference	Study Location	Sample Collection Year	Age	Selection	RV-Positive Samples/Total Samples	RV + Samples Attempted VP2/VP4 Amplification	Included in Meta-Analysis (A/B/C)	% Species Distribution (A/B/C)
Daleno et al., 2013 [167]	Italy	2007–2012 (November–April)	Children (0–14 years)	Hospitalized CAP	198/643 (30.7%)	198/198 (100%)	151 78/14/59	51.7/9.3/39
Espinola et al. 2013 [168]	Paraguay	May 2010–December 2011	Children (mean 8 months)	Hospitalized Acute LRTI	34/101 (33.66%)	34/34 (100%)	18 (13/1/4)	72/5.5/22
Pierangeli et al. 2013 [55]	Italy	2010	Children	Hospitalized RV + samples	90 *	73/90 (81%)	72 (40/5/27)	55/7/38
Sansone et al. 2013 [156]	Sweden	November 2006–September 2010	Children and Adults (51% < 10 years)	Hospitalized ARI	1840/11468 (16%)	170/1840 (9%)	106 (64/11/31)	56/9.6/32.4
Kiyota et al. 2014 [169]	Japan	January 2011–November 2012	Children and Adults (median 3 years)	ARI	96/904 (10.6%)	96/96 (100%)	78 (58/4/16)	60.4/4.2/35.4
Marcone et al. 2014 [48]	Argentina	June 2008–May 2010	Children (median 1 year)	Hospitalized and non-hospitalized ARI/ LRTI/URTI	252/620 (40.6%)	45/45 (100%)	41 (21/1/19)	46.6/2/42.2
Bruning et al. 2015 [170]	The Netherlands	November 2009–December 2012	Children (median 0.7–1.5 years)	Hospitalized and outpatients RV + samples	120/120 (100%)	120/120 (100%)	107 (63/4/40)	55/9/35
Jacobs et al. 2015 [171]	USA	April 2012–March 2013	Adults (65% > 50 years)	Hematologic malignancy	110 *	102/110 (92%)	66 (46/12/8)	64/12/21
L’Huillier et al. 2015 [172]	Tanzania	April–December 2008	Children (median 13.9 months)	Outpatients fever ≥ 38 degrees	244/1005 (24.2%)	244/244 (100%)	226 (119/39/68)	52/17/31
Naughtin et al. 2015 [54]	Cambodia	June 2007–December 2009	Children and Adults (60% < 5 years)	Hospitalized ILI /SARI	455/4170 (10.9%)	88/455 (19.3%)	60 (36/10/14)	60/16.6/28.3
Richter et al. 2015 [51]	Cyprus	November 2010–October 2013	Children (median 1.25 years)	ARI	116/485 (23.91%)	116/116 (100%)	68 (36/5/27)	54/12/35
Fall et al. 2016 [173]	Senegal	January 2012–December 2014	Children and Adults (median 4 years)	Outpatient ILI	1415/4194	150/1415 (10.6%)	87 (62/2/23)	57.9/5.3/36.8
Milanoi et al. 2016 [174]	Kenya	2008	Children and Adults (median 1.8 years)	Outpatient ILI	130/517 (25%)	37/130 (28%)	26 (14/3/9)	54/12/35
Tran et al. 2016 [158]	Vietnam	April 2010–May 2011	Children (mean 9 months)	Hospitalized ARI	325/1082 (30%)	58/325 (17.8%)	58 (44/0/14)	75.9/0/24.1
Van der Linden et al. 2016 [49]	The Netherlands	2007–2012	Children (median 1.6 years)	Samples sent to diagnostic	1102/6258 (17.6%)	745/1102 (67.9%)	587 (309/56/222)	52.4/11.3/36.2
Saraya et al. 2017 [109]	Japan	August 2012–May 2015	Adults (median 56 years)	Asthma attack	24/106 (22.6%)	24/24 (100%)	21 (12/1/8)	50/4.2/45.8
Andres et al. 2018 [157]	Spain	October 2014–May 2017	Children and adults (median 5 years)	RTI suspicion	2615/19957 (13.1%)	1771/2615 (68%)	1545 (948/90/507)	63/6/31
Morobe et al. 2018 [50]	Kenya	December 2015–November 2016	Children and adults (median 2 years)	Outpatient ARI	1057/5744 (18.4%)	817/1057 (77.3%)	776 (359/67/350)	44/8.2/47.8
Ng et al. 2018 [36]	Malaysia	February 2012–May 2014	Children and Adults (median 38 years)	Outpatients ARI	976/3935 (24.8%)	976/976 (100%)	111 ** (54/16/41)	49/13/38
Zhao et al. 2018 [53]	China	2013–2015	Children (median 1 year)	Hospitalized SARI	280/1003 (28%)	280/280 (100%)	217 (140/21/56)	50/7.5/20
Baillie et al. 2019 [34]	Mali, South Africa and Zambia	August 2011–August 2013	Children (<5 years)	Controls and Acute pneumonia (hospitalized)	901/4404 (20.4%)	836/901 (92.7%)	757 (357/67/333)	46.5/8.5/45
Hung et al. 2019 [124]	Taiwan	January 2013–December 2014	Children and Adults (median 3.8 years)	Hospitalized ARI	76/487	76/76	47 ** (29/4/14)	54/7.9/38
Ko et al. 2019 [102]	Hong Kong	August 2016–July 2017	Adult (median 48–77.4)	Exacerbation COPD and Asthma	38/603	38/38	38 (21/4/13)	55/10/34
Kuypers et al. 2019 [175]	Nepal	December 2012–April 2014	Infants (0–180 days)	Resp. symptoms not hospitalized	547/609	285/647	265 (183/26/64)	68/6.3/26
Arden et al. 2020 [31]	Australia	2001	Children and Adults (median 1.5 years)	ARI	266/1179	266/266	249 (11/108/17)	53/4/42
Linster et al. 2020 [176]	Singapore	2007–2013	Adults (median 31 years)	Febrile illness	236/2950	163/236	131 (92/17/22)	65.2/16.3/12
Luka et al. 2020 [159]	Kenya	May 2017–April 2018	348 children and 4 adults (school)	ARI	307/1859	253/307	241 (134/46/61)	53/18.2/28.9
Adam et al. 2021 [29]	Australia	2006–2009	Children (under 18 years)	Mixed previous cohorts	91	43/91	43 (24/15/4)	48/8/44
Golke et al. 2021 [30]	Germany	2013–2017	Adults (mean 54.8 years)	URTI/LRTI	506/11650	410/506	284 (173/36/75)	60.9/12.7/26.4
Haddad-Boubaker et al. 2021 [101]	Tunisia	September 2015–December 2017	Children (median 2 months)	Hospitalized SARI, ICU	57/271	49/57	49 (32/3/14)	63.3/6.1/30.6
Li et al. 2021 [32]	China	August 2018–December 2019	Children (median 16–21 months)	Hospitalized ARI	42/655	40/49	17/0/23	45/0/55
